# Supplementary material for: Normative Data for Ten Neuropsychological Tests for the Guatemalan Pediatric Population Updated to Account for Vulnerability
Source: Brain Sci. 2021 Jun 25;11(7):842. doi: 10.3390/brainsci11070842 (PMC8301765; doi:10.3390/brainsci11070842)
Supplement: Supplementary file 1 [file brainsci-11-00842-s001.zip › Supplementary material 1.pdf]

Normative data for ten neuropsychological tests for the Guatemalan pediatric population updated to account for vulnerability

Supplementary material 1: Tables

Table S1. Sample description

	Sample ages (years)			
	From 6 to 11		From 12 to 17	
	V	NV	V	NV
N	87	129	57	74
Medium age (SD)	8.41(1.87)	9.19(1.44)	14.96(1.59)	13.35(1.64)
% Female	48.3	43.4	50.9	52.7

SD: standard deviation, V: vulnerable, NV: nonvulnerable

Table S2. Multivariant model for the ROCF test

Neuropsychological Tests	Predictors	B	Standard error	β	*t*	*p*-value	Adjusted R^2
ROCF Copy	(constant)	32.147	0.57	-	56.394	<.001	.503
	Age	1.956	0.12	0.828	16.246	<.001	
	Age^2	-0.249	0.03	-0.352	-8.367	<.001	
	AgeXvulnerability	-0.628	0.183	-0.169	-3.422	.001	
	Vulnerability	-0.318	0.585	-0.022	-0.545	.586	
ROCF Memory	(constant)	19.72	0.481	-	41.011	<.001	.392
	Age	1.777	0.121	0.646	14.694	<.001	
	MLPE	0.245	0.075	0.138	3.289	.001	
	Age^2	-0.132	0.036	-0.161	-3.656	<.001	

ROCF: Rey Osterrieth Complex Figure Test, and MLPE: Mean level of parental education

Normative data for ten neuropsychological tests for the Guatemalan pediatric population updated to account for vulnerability

Table S3. Multivariant model for the TAMV-I test

Neuropsychological Tests	Predictors	B	Standard error	β	*t*	*p*-value	Adjusted R^2
TAMV-I immediate	(Constant)	34.679	0.635	-	38.389	<.001	.227
	Gender	-2.381	0.726	-0.156	-3.279	.001	
	Age	1.090	0.125	0.431	8.649	<.001	
	MLPE	0.591	0.127	0.363	4.663	<.001	
	Age^2	-0.132	0.041	-0.174	-3.190	.002	
	Vulnerability	-0.754	0.868	-0.049	-0.869	.386	
	MLPEXvulnerability	-0.581	0.177	-0.247	-3.286	.001	
TAMV-I delayed	(Constant)	10.017	0.265	-	37.767	<.001	.290
	Vulnerability	-0.124	0.255	-0.026	-0.485	.628	
	Gender	-0.789	0.213	-0.169	-3.703	<.001	
	Age	0.375	0.037	0.484	10.176	<.001	
	MLPE	0.144	0.037	0.289	3.865	<.001	
	Age^2	-0.048	0.012	-0.206	-3.932	<.001	
	AgeXMLPE	-0.028	0.007	-0.183	-4	<.001	
	MLPEXvulnerability	-0.147	0.052	-0.205	-2.835	.005	
TAMV-I recognition	(Constant)	11.346	0.09		125.517	<.001	.151
	Age	0.163	0.023	0.369	7.138	<.001	
	MLPE	0.038	0.014	0.135	2.719	.007	
	Age^2	-0.022	0.007	-0.166	-3.209	.001	
	AgeXMLPE	-0.013	0.004	-0.153	-3.081	.002	

TAMV-I: Learning and Verbal Memory Test, and MLPE: Mean level of parental education

Table S4. Multivariant model for the SDMT test

Neuropsychological Tests	Predictors	B	Standard error	β	*t*	*p*-value	Adjusted R^2
SDMT	(Constant)	31.731	0.9	-	35.269	<.001	.675
	Vulnerability	3.084	0.962	0.117	3.207	.001	
	Age	3.646	0.145	0.835	25.231	<.001	
	MLPE	0.44	0.096	0.158	4.559	<.001	
	Age^2	-0.142	0.046	-0.109	-3.096	.002	

SDMT: Symbol Digit Modalities Tests, and MLPE: Mean level of parental education

Table S5. Multivariant model for the d2 concentration index test

Neuropsychological Tests	Predictors	B	Standard error	β	*t*	*p*-value	Adjusted R^2
d2 Concentration Index	(Constant)	92.921	2.912	-	31.909	<.001	.551
	Vulnerability	26.775	3.956	0.275	6.768	<.001	
	Age	10.808	0.592	0.677	18.261	<.001	
	MLPE	1.626	0.418	0.158	3.888	<.001	
d2 Total number of responses	(Constant)	304.578	5.988	-	50.866	<.001	.502
	Vulnerability	49.990	7.828	0.249	6.386	<.001	
	Age	22.562	1.284	0.685	17.57	<.001	
d2 Total correct	(Constant)	107.647	2.589	-	41.577	<.001	.520
	Vulnerability	21.557	3.517	0.258	6.129	<.001	
	Age	9.093	0.526	0.662	17.281	<.001	
	MLPE	1.367	0.372	0.152	3.622	<.001	
d2 Total effectiveness	(Constant)	269.049	5.797	-	46.408	<.001	.585

	Vulnerability	52.831	7.876	0.262	6.708	<.001
	Age	23.643	1.178	0.715	20.066	<.001
	MLPE	2.898	0.833	0.136	3.480	.001

MLPE: Mean level of parental education

Table S6. Multivariant model for the PPVT-III test

Neuropsychological Tests	Predictors	B	Standard error	β	*t*	*p*-value	Adjusted R^2
PPVT-III	(Constant)	89.974	1.64	-	54.862	<.001	.647
	Vulnerability	18.672	2.118	0.315	8.814	<.001	
	Age	6.568	0.316	0.674	20.769	<.001	
	MLPE	2.27	0.328	0.365	6.929	<.001	
	MLPEXvulnerability	-1.353	0.447	-0.15	-3.028	.003	

PPVT-III: Peabody Picture Vocabulary Test, and MLPE: Mean level of parental education

Table S7. Multivariant model for the Token test

Neuropsychological Tests	Predictors	B	Standard error	β	*t*	*p*-value	Adjusted R^2
Token	(Constant)	30.176	0.314		96.15	<.001	.396
	Vulnerability	1.084	0.336	0.16	3.221	.001	
	Age	0.626	0.049	0.565	12.913	<.001	
	MLPE	0.152	0.033	0.213	4.545	<.001	
	Age^2	-0.068	0.016	-0.206	-4.394	<.001	

MLPE: Mean level of parental education

Table S8. Multivariant model for the verbal fluency test

Neuropsychological Tests	Predictors	B	Standard error	β	*t*	*p*-value	Adjusted R^2
Verbal Fluency Tests-Phonolog. A	(Constant)	7.480	0.158	-	47.453	<.001	.324
	Age	0.648	0.053	0.547	12.343	<.001	
	MLPE	0.145	0.034	0.19	4.292	<.001	
Verbal Fluency Tests-Phonolog. F	(Constant)	5.598	0.243	-	23.045	<.001	.393
	Vulnerability	1.544	0.317	0.205	4.865	<.001	
	Age	0.745	0.052	0.603	14.315	<.001	
Verbal Fluency Tests-Phonolog. S	(Constant)	7.388	0.164	-	44.955	<.001	.321
	Age	0.678	0.055	0.551	12.379	<.001	
	MLPE	0.117	0.035	0.149	3.335	.001	
Verbal Fluency Tests-Semant. Animals	(Constant)	14.443	0.193	-	74.8	<.001	.383
	Age	0.887	0.064	0.584	13.827	<.001	
	MLPE	0.221	0.041	0.226	5.352	<.001	
Verbal Fluency Tests-Semant. Fruits	(Constant)	11.148	0.185	-	60.260	<.001	.314
	Age	0.586	0.047	0.584	12.538	<.001	
	Age^2	-0.046	0.014	-0.154	-3.309	.001	

MLPE: Mean level of parental education

Table S9. Multivariant model for the Stroop test

Neuropsychological Tests	Predictors	B	Standard error	β	*t*	*p*-value	Adjusted R^2
Stroop word	(Constant)	68.927	1.664	-	41.415	<.001	.547
	Vulnerability	7.883	1.809	0.195	4.359	<.001	
	Age	6.850	0.448	0.979	15.279	<.001	
	MLPE	0.561	0.188	0.129	2.983	.003	
	Age2	-0.498	0.096	-0.250	-5.194	<.001	
	AgeXvulnerability	-2.293	0.590	-0.219	-3.884	<.001	
Stroop color	(Constant)	51.609	0.587	-	87.866	<.001	.414
	Age	3.042	0.209	0.646	14.533	<.001	
	MLPE	0.381	0.130	0.130	2.932	.004	
Stroop color and word	(Constant)	29.252	0.431		67.938	<.001	.305
	Age	1.756	0.152	0.553	11.525	<.001	
Stroop interference	(Constant)	1.72	0.755	-	2.277	.023	.085
	Vulnerability	-3.547	0.934	-0.233	-3.798	<.001	
	Age	-0.687	0.208	-0.26	-3.295	<.001	
	MLPE	-0.362	0.146	-0.221	-2.477	.014	
	AgeXvulnerability	0.862	0.302	0.218	2.857	.005	
	MLPEXvulnerability	0.619	0.198	0.263	3.126	.002	

MLPE: Mean level of parental education

Table S10. Multivariant model for the TMT A-B test

Neuropsychological Tests	Predictors	B	Standard error	β	*t*	*p*-value	Adjusted R^2
TMT-A	(Constant)	37.097	1.787	-	20.757	<.001	.390
	Vulnerability	-9.673	1.916	-0.252	-5.05	<.001	
	Age	-2.892	0.374	-0.456	-7.723	<.001	
	MLPE	-0.99	0.193	-0.245	-5.128	<.001	
	Age^2	0.762	0.091	0.404	8.381	<.001	
	AgeXvulnerability	1.984	0.551	0.203	3.602	<.001	.298
TMT-B	(Constant)	66.897	3.063	-	21.838	<.001	
	Vulnerability	-18.341	3.195	-0.28	-5.741	<.001	
	Age	-3.925	0.567	-0.358	-6.918	<.001	
	Age^2	1.418	0.167	0.45	8.484	<.001	

TMT A-B: Trail Making Test A & B, and MLPE: Mean level of parental education

Table S11. Multivariant model for the M-WCST test

Neuropsychological Tests	Predictors	B	Standard error	β	*t*	*p*-value	Adjusted R^2
M-WCST correct categories	(Constant)	4.563	0.134	-	34.063	<.001	.206
	Vulnerability	-0.509	0.175	-0.14	-2.906	.004	
	Age	0.257	0.029	0.431	8.956	<.001	
M-WCST perseverative errors	(Constant)	2.494	0.496	-	5.025	<.001	.247
	Vulnerability	2.150	0.596	0.250	3.608	<.001	
	Age	-0.843	0.092	-0.598	-9.198	<.001	
	Age^2	0.153	0.030	0.365	5.053	<.001	
	AgeXvulnerability	0.491	0.141	0.222	3.484	<.001	
	Age^2Xvulnerability	-0.233	0.044	-0.396	-5.329	<.001	
M-WCST total errors	(Constant)	9.200	1.120	-	8.217	<.001	.271
	Vulnerability	6.511	1.333	0.326	4.884	<.001	
	Age	-1.645	0.159	-0.503	-10.353	<.001	
	Age^2	0.310	0.067	0.318	4.592	<.001	
	Age^2Xvulnerability	-0.409	0.096	-0.299	-4.266	<.001	

M-WCST: Modified Wisconsin Card Shorting Test, MLPE: Mean level of parental education

Normative data for ten neuropsychological tests for the Guatemalan pediatric population updated to account for vulnerability

Table S12. Standard deviations of the ten neuropsychological tasks' residual values

**Neuropsychological test**	**Predictive values ($\hat{Y}_i$)** [1]	**SD*e***
ROCF copy	≤ 27.54	6.82
	27.54 – 31.51	5.38
	31.51-33.05	4.12
	> 33.05	2.66
ROCF memory	All values	6.47
TAMV-I immediate	All values	6.65
TAMV-I delayed	≤ 8.3	2.13
	8.301 - 9.179	2.28
	9.18 - 9.876	1.84
	> 9.877	1.44
TAMV-I recognition	≤ 10.897	1.64
	10.898 - 11.293	1.34
	11.294 - 11.559	0.98
	> 11.56	0.74
d2 concentration index	All values	32.04
d2 total responses	≤ 289.9906	69.57
	289.9907 – 335.1146	59.09
	335.1147- 381.3439	65.35
	> 381.344	83.83
d2 total correct	All values	28.41
d2 total effectiveness TOT	≤ 253.0497	56.58
	253.0498 - 297.2198	54.66
	297.2199 - 355.5761	70.36

	> 355.5762	71.65
PPVT-III	≤ 86.755	15.15
	86.756 - 101.249	19.12
	101.25 - 114.379	20.14
	> 114.379	15.24
Token	≤ 29.144	3.41
	29.145 - 30.517	2.59
	30.518 - 31.48	2.24
	> 31.481	1.88
Phonol.Verbal fluency F	All values	2.89
Phonol.Verbal fluency A	All values	2.92
Phonol.Verbal fluency S	All values	3.05
Sem.Verbal fluency Animals	≤ 12.327	3.33
	12.328 - 14.027	3.40
	14.028 - 16.206	3.50
	> 16.207	4.12
Sem.Verbal fluency Fruits	All values	2.51
Stroop Word	≤ 61.2825	16.48
	61.283 - 72.531	11.49
	72.5306 - 83.0317	13.48
	> 83.0318	12.12
Stroop color	≤ 45.0257	7.39
	45.0258 - 50.3489	10.36
	50.349 - 57.3263	11.04
	> 57.3264	10.35
Stroop color and word	All values	7.42
Stroop interference	All values	7.01
TMT-A	≤ 28.964	9.75
	28.965 - 34.408	10.79
	34.409 - 44.688	16.23
	> 44.689	19.91
TMT-B	≤ 50.449	16.11
	50.45 - 65.687	23.15
	65.688 - 79.44	29.76
	> 79.441	35.45

M-WCST correct responses	≤ 3.588	1.80
	3.589 - 4.102	1.66
	4.103 - 4.653	1.59
	> 4.654	1.24
M-WCST perseverative err.	≤ 2.649	2.31
	2.6491 - 4.482	3.02
	4.4821 - 5.0004	4.06
	> 5.0005	4.87
M-WCST total errors	≤ 9.461	7.20
	9.4611 - 15.1837	7.55
	15.1838 - 18.3672	8.86
	> 18.3673	10.02

[1] Predictive values by using the *Formula 1* (methods' section) in each neuropsychological test
