# Supplementary material for: Normative Data for Ten Neuropsychological Tests for the Guatemalan Pediatric Population Updated to Account for Vulnerability
Source: Brain Sci. 2021 Jun 25;11(7):842. doi: 10.3390/brainsci11070842 (PMC8301765; doi:10.3390/brainsci11070842)
Supplement: Supplementary file 1 [file brainsci-11-00842-s001.zip › Supplementary material 2.pdf]

Normative data for ten neuropsychological tests for the Guatemalan pediatric population updated to account for vulnerability

Table S13. Transformation from values (z_i) to percentile scores in ROCF copy task.

PC	ROCF-copy	PC	ROCF-copy	PC	ROCF-copy	PC	ROCF-copy
		**25**	-0,447	**50**	0,226	**75**	0,709
**1**	-3,957	**26**	-0,408	**51**	0,226	**76**	0,715
**2**	-2,879	**27**	-0,383	**52**	0,228	**77**	0,715
**3**	-2,403	**28**	-0,342	**53**	0,234	**78**	0,715
**4**	-2,035	**29**	-0,296	**54**	0,242	**79**	0,720
**5**	-1,877	**30**	-0,267	**55**	0,259	**80**	0,720
**6**	-1,707	**31**	-0,256	**56**	0,261	**81**	0,766
**7**	-1,630	**32**	-0,219	**57**	0,263	**82**	0,817
**8**	-1,529	**33**	-0,148	**58**	0,263	**83**	0,818
**9**	-1,413	**34**	-0,112	**59**	0,288	**84**	0,833
**10**	-1,289	**35**	-0,107	**60**	0,316	**85**	0,924
**11**	-1,190	**36**	-0,084	**61**	0,354	**86**	0,927
**12**	-1,132	**37**	-0,033	**62**	0,406	**87**	0,954
**13**	-1,021	**38**	-0,016	**63**	0,447	**88**	0,959
**14**	-0,979	**39**	0,002	**64**	0,469	**89**	0,963
**15**	-0,967	**40**	0,004	**65**	0,472	**90**	1,003
**16**	-0,861	**41**	0,012	**66**	0,472	**91**	1,003
**17**	-0,798	**42**	0,043	**67**	0,472	**92**	1,006
**18**	-0,762	**43**	0,069	**68**	0,489	**93**	1,007
**19**	-0,745	**44**	0,077	**69**	0,505	**94**	1,048
**20**	-0,742	**45**	0,106	**70**	0,555	**95**	1,089
**21**	-0,671	**46**	0,142	**71**	0,628	**96**	1,140
**22**	-0,585	**47**	0,171	**72**	0,631	**97**	1,214
**23**	-0,541	**48**	0,199	**73**	0,635	**98**	1,307
**24**	-0,487	49	0,226	**74**	0,662	**99**	1,415

Table S14. Transformation from values (z_i) to percentile scores in delayed and recognition verbal memory tasks.

PC	Delayed verbal memory	Recog. verbal memory	PC	Delayed verbal memory	Recog. verbal memory	PC	Delayed verbal memory	Recog. verbal memory	PC	Delayed verbal memory	Recog. verbal memory
			**25**	-0,622	-0,423	**50**	0,060	0,472	**75**	0,771	0,611
**1**	-3,245	-3,479	**26**	-0,573	-0,396	**51**	0,074	0,477	**76**	0,785	0,614
**2**	-2,740	-3,054	**27**	-0,523	-0,385	**52**	0,088	0,491	**77**	0,794	0,615
**3**	-2,155	-2,463	**28**	-0,481	-0,345	**53**	0,099	0,495	**78**	0,812	0,624
**4**	-1,970	-2,345	**29**	-0,466	-0,308	**54**	0,134	0,496	**79**	0,822	0,628
**5**	-1,829	-2,274	**30**	-0,456	-0,271	**55**	0,165	0,501	**80**	0,849	0,629
**6**	-1,731	-2,221	**31**	-0,428	-0,193	**56**	0,201	0,502	**81**	0,869	0,653
**7**	-1,555	-2,098	**32**	-0,419	-0,133	**57**	0,228	0,504	**82**	0,882	0,661
**8**	-1,418	-1,714	**33**	-0,384	-0,086	**58**	0,254	0,511	**83**	0,907	0,668
**9**	-1,359	-1,527	**34**	-0,339	-0,060	**59**	0,279	0,520	**84**	0,918	0,677
**10**	-1,316	-1,451	**35**	-0,323	-0,004	**60**	0,322	0,529	**85**	0,939	0,683
**11**	-1,181	-1,341	**36**	-0,303	0,040	**61**	0,353	0,534	**86**	0,959	0,692
**12**	-1,065	-1,181	**37**	-0,232	0,120	**62**	0,369	0,540	**87**	0,986	0,702
**13**	-1,029	-0,998	**38**	-0,191	0,160	**63**	0,373	0,542	**88**	1,143	0,713
**14**	-0,981	-0,956	**39**	-0,159	0,184	**64**	0,394	0,550	**89**	1,172	0,724
**15**	-0,889	-0,932	**40**	-0,141	0,247	**65**	0,420	0,555	**90**	1,192	0,733
**16**	-0,868	-0,898	**41**	-0,123	0,303	**66**	0,460	0,561	**91**	1,248	0,753
**17**	-0,852	-0,846	**42**	-0,112	0,341	**67**	0,505	0,564	**92**	1,266	0,755
**18**	-0,834	-0,795	**43**	-0,103	0,387	**68**	0,519	0,577	**93**	1,296	0,801
**19**	-0,796	-0,779	**44**	-0,079	0,418	**69**	0,563	0,580	**94**	1,350	0,826
**20**	-0,791	-0,709	**45**	-0,075	0,429	**70**	0,614	0,583	**95**	1,421	0,859
**21**	-0,774	-0,559	**46**	-0,061	0,449	**71**	0,657	0,591	**96**	1,481	0,899
**22**	-0,723	-0,527	**47**	-0,003	0,450	**72**	0,687	0,592	**97**	1,517	0,944
**23**	-0,667	-0,495	**48**	0,021	0,458	**73**	0,712	0,595	**98**	1,565	0,991
**24**	-0,655	-0,457	**49**	0,046	0,462	**74**	0,757	0,609	**99**	1,710	1,064

Table S15. Transformation from values (z_i) to percentile scores in phonological verbal fluency tasks.

PC	Phonol. Fluency A	Phonol. Fluency F	Phonol. Fluency S	PC	Phonol. Fluency A	Phonol. Fluency F	Phonol. Fluency S	PC	Phonol. Fluency A	Phonol. Fluency F	Phonol. Fluency S	PC	Phonol. Fluency A	Phonol. Fluency F	Phonol. Fluency S
				**25**	-1,286	-0,702	-0,734	**50**	-0,230	-0,095	-0,131	**75**	1,224	0,643	0,662
**1**	-4,186	-2,015	-2,055	**26**	-1,215	-0,698	-0,725	**51**	-0,177	-0,089	-0,113	**76**	1,343	0,680	0,698
**2**	-3,602	-1,900	-1,822	**27**	-1,181	-0,691	-0,670	**52**	-0,153	-0,016	-0,099	**77**	1,391	0,695	0,790
**3**	-3,385	-1,744	-1,684	**28**	-1,122	-0,649	-0,654	**53**	-0,100	-0,016	-0,079	**78**	1,443	0,731	0,824
**4**	-3,167	-1,561	-1,548	**29**	-1,099	-0,628	-0,622	**54**	-0,072	-0,009	-0,065	**79**	1,475	0,761	0,836
**5**	-2,954	-1,411	-1,429	**30**	-1,076	-0,617	-0,603	**55**	0,009	0,034	-0,032	**80**	1,643	0,776	0,860
**6**	-2,696	-1,387	-1,356	**31**	-1,036	-0,617	-0,573	**56**	0,055	0,072	0,016	**81**	1,698	0,890	0,883
**7**	-2,549	-1,296	-1,292	**32**	-0,986	-0,617	-0,532	**57**	0,085	0,102	0,060	**82**	1,796	0,937	0,915
**8**	-2,421	-1,248	-1,242	**33**	-0,977	-0,582	-0,510	**58**	0,156	0,133	0,115	**83**	1,857	0,970	0,961
**9**	-2,384	-1,206	-1,221	**34**	-0,895	-0,537	-0,490	**59**	0,239	0,160	0,140	**84**	1,926	1,025	1,021
**10**	-2,289	-1,130	-1,178	**35**	-0,852	-0,529	-0,435	**60**	0,273	0,168	0,153	**85**	1,982	1,103	1,086
**11**	-2,263	-1,050	-1,156	**36**	-0,817	-0,529	-0,427	**61**	0,316	0,207	0,188	**86**	2,089	1,111	1,161
**12**	-2,171	-1,045	-1,128	**37**	-0,753	-0,500	-0,406	**62**	0,324	0,248	0,213	**87**	2,158	1,193	1,226
**13**	-2,100	-1,042	-1,108	**38**	-0,700	-0,442	-0,394	**63**	0,347	0,253	0,238	**88**	2,235	1,309	1,291
**14**	-2,068	-1,035	-1,047	**39**	-0,643	-0,367	-0,381	**64**	0,487	0,284	0,247	**89**	2,294	1,369	1,340
**15**	-1,957	-0,971	-0,975	**40**	-0,539	-0,353	-0,332	**65**	0,533	0,328	0,264	**90**	2,423	1,452	1,385
**16**	-1,848	-0,962	-0,949	**41**	-0,514	-0,353	-0,316	**66**	0,657	0,336	0,303	**91**	2,522	1,509	1,447
**17**	-1,748	-0,943	-0,933	**42**	-0,498	-0,331	-0,285	**67**	0,737	0,372	0,318	**92**	2,620	1,613	1,549
**18**	-1,724	-0,895	-0,897	**43**	-0,466	-0,281	-0,259	**68**	0,815	0,385	0,391	**93**	2,825	1,628	1,750
**19**	-1,667	-0,874	-0,877	**44**	-0,390	-0,273	-0,239	**69**	0,872	0,416	0,416	**94**	2,950	1,773	1,789
**20**	-1,618	-0,872	-0,841	**45**	-0,360	-0,265	-0,222	**70**	0,901	0,472	0,439	**95**	3,258	1,829	1,881
**21**	-1,532	-0,844	-0,823	**46**	-0,347	-0,240	-0,187	**71**	0,929	0,497	0,483	**96**	3,618	1,972	1,931
**22**	-1,499	-0,786	-0,812	**47**	-0,324	-0,183	-0,176	**72**	1,053	0,504	0,533	**97**	3,884	2,108	1,983
**23**	-1,453	-0,786	-0,791	**48**	-0,301	-0,137	-0,166	**73**	1,151	0,548	0,586	**98**	4,556	2,237	2,097
**24**	-1,365	-0,761	-0,743	**49**	-0,283	-0,097	-0,157	**74**	1,188	0,598	0,601	**99**	6,072	2,446	2,478

Table S16. Transformation from values (z_i) to percentile scores in semantic verbal fluency tasks.

	Fluency animals	Fluency fruits		Fluency animals	Fluency fruits		Fluency animals	Fluency fruits		Fluency animals	Fluency fruits
			**25**	-0,631	-0,707	**50**	-0,062	-0,065	**75**	0,548	0,638
**1**	-1,977	-2,429	**26**	-0,618	-0,707	**51**	-0,053	-0,053	**76**	0,597	0,703
**2**	-1,861	-2,052	**27**	-0,599	-0,652	**52**	0,004	-0,034	**77**	0,654	0,810
**3**	-1,805	-1,676	**28**	-0,589	-0,597	**53**	0,017	0,010	**78**	0,729	0,822
**4**	-1,636	-1,644	**29**	-0,579	-0,500	**54**	0,035	0,027	**79**	0,785	0,822
**5**	-1,550	-1,560	**30**	-0,532	-0,499	**55**	0,057	0,027	**80**	0,823	0,854
**6**	-1,466	-1,509	**31**	-0,509	-0,499	**56**	0,089	0,027	**81**	0,921	0,884
**7**	-1,410	-1,483	**32**	-0,496	-0,466	**57**	0,135	0,052	**82**	0,979	0,899
**8**	-1,369	-1,352	**33**	-0,474	-0,451	**58**	0,144	0,086	**83**	1,010	0,939
**9**	-1,286	-1,279	**34**	-0,454	-0,431	**59**	0,166	0,103	**84**	1,023	0,991
**10**	-1,237	-1,167	**35**	-0,433	-0,401	**60**	0,196	0,143	**85**	1,067	1,030
**11**	-1,202	-1,105	**36**	-0,406	-0,385	**61**	0,210	0,175	**86**	1,123	1,091
**12**	-1,147	-1,091	**37**	-0,389	-0,371	**62**	0,233	0,240	**87**	1,148	1,130
**13**	-1,117	-1,041	**38**	-0,366	-0,371	**63**	0,239	0,297	**88**	1,176	1,199
**14**	-1,075	-0,962	**39**	-0,346	-0,317	**64**	0,274	0,315	**89**	1,244	1,224
**15**	-1,040	-0,898	**40**	-0,323	-0,310	**65**	0,291	0,345	**90**	1,311	1,269
**16**	-0,988	-0,897	**41**	-0,311	-0,310	**66**	0,303	0,367	**91**	1,369	1,319
**17**	-0,929	-0,897	**42**	-0,286	-0,279	**67**	0,328	0,405	**92**	1,463	1,431
**18**	-0,893	-0,879	**43**	-0,255	-0,255	**68**	0,337	0,422	**93**	1,572	1,603
**19**	-0,871	-0,799	**44**	-0,202	-0,250	**69**	0,353	0,424	**94**	1,627	1,674
**20**	-0,819	-0,785	**45**	-0,188	-0,189	**70**	0,416	0,480	**95**	1,744	1,752
**21**	-0,789	-0,769	**46**	-0,178	-0,143	**71**	0,458	0,486	**96**	1,940	1,888
**22**	-0,767	-0,769	**47**	-0,151	-0,102	**72**	0,484	0,494	**97**	2,048	2,027
**23**	-0,746	-0,767	**48**	-0,103	-0,102	**73**	0,500	0,526	**98**	2,349	2,288
**24**	-0,687	-0,746	**49**	-0,091	-0,092	**74**	0,529	0,541	**99**	2,835	2,682

Table S17. Transformation from values (z_i) to percentile scores in word word/color, and interference Stroop tasks.

PC	Stroop-Word	Stroop Word/color	Stroop interf.	PC	Stroop-Word	Stroop Word/color	Stroop interf.	PC	Stroop-Word	Stroop Word/color	Stroop interf.	PC	Stroop-Word	Stroop Word/color	Stroop interf.
				**25**	-0,623	-0,652	-0,624	**50**	0,085	-0,009	-0,002	**75**	0,085	0,587	0,553
**1**	-2,682	-2,365	-2,457	**26**	-0,596	-0,613	-0,557	**51**	0,103	-0,009	0,007	**76**	0,103	0,630	0,572
**2**	-1,987	-1,992	-2,141	**27**	-0,579	-0,580	-0,529	**52**	0,114	-0,009	0,027	**77**	0,114	0,650	0,583
**3**	-1,897	-1,775	-1,886	**28**	-0,529	-0,547	-0,513	**53**	0,177	0,020	0,035	**78**	0,177	0,664	0,617
**4**	-1,857	-1,450	-1,691	**29**	-0,505	-0,523	-0,499	**54**	0,200	0,043	0,050	**79**	0,200	0,693	0,652
**5**	-1,800	-1,391	-1,409	**30**	-0,484	-0,510	-0,464	**55**	0,233	0,082	0,078	**80**	0,233	0,726	0,659
**6**	-1,625	-1,372	-1,379	**31**	-0,444	-0,482	-0,422	**56**	0,257	0,093	0,111	**81**	0,257	0,776	0,706
**7**	-1,548	-1,345	-1,330	**32**	-0,429	-0,449	-0,398	**57**	0,271	0,102	0,122	**82**	0,271	0,822	0,749
**8**	-1,476	-1,276	-1,269	**33**	-0,378	-0,437	-0,379	**58**	0,304	0,124	0,134	**83**	0,304	0,864	0,796
**9**	-1,387	-1,253	-1,210	**34**	-0,336	-0,414	-0,372	**59**	0,342	0,144	0,138	**84**	0,342	0,876	0,820
**10**	-1,369	-1,208	-1,193	**35**	-0,294	-0,413	-0,363	**60**	0,360	0,165	0,154	**85**	0,360	0,966	0,858
**11**	-1,356	-1,118	-1,137	**36**	-0,260	-0,394	-0,341	**61**	0,384	0,188	0,190	**86**	0,384	1,000	0,907
**12**	-1,304	-1,050	-1,087	**37**	-0,233	-0,355	-0,327	**62**	0,399	0,201	0,235	**87**	0,399	1,051	0,964
**13**	-1,229	-1,020	-1,059	**38**	-0,221	-0,318	-0,293	**63**	0,418	0,224	0,255	**88**	0,418	1,104	0,979
**14**	-1,151	-0,985	-1,034	**39**	-0,199	-0,311	-0,283	**64**	0,443	0,249	0,282	**89**	0,443	1,146	1,003
**15**	-1,065	-0,958	-0,962	**40**	-0,176	-0,281	-0,253	**65**	0,447	0,267	0,303	**90**	0,447	1,267	1,026
**16**	-1,040	-0,901	-0,925	**41**	-0,135	-0,249	-0,217	**66**	0,469	0,303	0,341	**91**	0,469	1,346	1,083
**17**	-0,910	-0,879	-0,908	**42**	-0,119	-0,245	-0,190	**67**	0,493	0,326	0,365	**92**	0,493	1,369	1,219
**18**	-0,868	-0,849	-0,880	**43**	-0,090	-0,229	-0,177	**68**	0,525	0,352	0,403	**93**	0,525	1,401	1,423
**19**	-0,851	-0,824	-0,803	**44**	-0,034	-0,202	-0,155	**69**	0,568	0,393	0,443	**94**	0,568	1,558	1,535
**20**	-0,818	-0,816	-0,771	**45**	0,008	-0,143	-0,140	**70**	0,597	0,413	0,481	**95**	0,597	1,706	1,829
**21**	-0,781	-0,754	-0,746	**46**	0,037	-0,120	-0,124	**71**	0,606	0,427	0,497	**96**	0,606	1,783	2,081
**22**	-0,739	-0,718	-0,727	**47**	0,053	-0,103	-0,103	**72**	0,636	0,443	0,524	**97**	0,636	1,965	2,252
**23**	-0,692	-0,687	-0,694	**48**	0,059	-0,041	-0,062	**73**	0,659	0,486	0,531	**98**	0,659	2,510	2,871

**24**	-0,676	-0,678	-0,678	**49**	0,074	-0,012	-0,039	**74**	0,676	0,493	0,539	**99**	0,676	3,280	3,135

Table S18. Transformation from values (z_i) to percentile scores in TMT tasks.

PC	TMT-A	TMT-B	PC	TMT-A	TMT-B	PC	TMT-A	TMT-B	PC	TMT-A	TMT-B
			**25**	-0,705	-0,718	**50**	-0,125	-0,131	**75**	0,618	0,466
**1**	-1,794	-1,933	**26**	-0,689	-0,703	**51**	-0,092	-0,117	**76**	0,659	0,503
**2**	-1,595	-1,607	**27**	-0,671	-0,676	**52**	-0,066	-0,109	**77**	0,686	0,539
**3**	-1,491	-1,567	**28**	-0,632	-0,662	**53**	-0,008	-0,095	**78**	0,715	0,562
**4**	-1,407	-1,485	**29**	-0,608	-0,643	**54**	0,001	-0,081	**79**	0,738	0,590
**5**	-1,370	-1,390	**30**	-0,572	-0,581	**55**	0,013	-0,057	**80**	0,812	0,620
**6**	-1,342	-1,322	**31**	-0,536	-0,576	**56**	0,031	-0,035	**81**	0,899	0,664
**7**	-1,287	-1,251	**32**	-0,506	-0,549	**57**	0,047	-0,024	**82**	0,969	0,783
**8**	-1,249	-1,183	**33**	-0,481	-0,516	**58**	0,072	-0,014	**83**	1,010	0,815
**9**	-1,194	-1,136	**34**	-0,463	-0,501	**59**	0,106	0,069	**84**	1,081	0,904
**10**	-1,152	-1,108	**35**	-0,447	-0,485	**60**	0,141	0,115	**85**	1,117	0,956
**11**	-1,139	-1,077	**36**	-0,439	-0,436	**61**	0,157	0,130	**86**	1,135	1,114
**12**	-1,116	-1,049	**37**	-0,423	-0,419	**62**	0,199	0,140	**87**	1,162	1,185
**13**	-1,073	-1,039	**38**	-0,392	-0,397	**63**	0,238	0,149	**88**	1,217	1,202
**14**	-1,055	-0,996	**39**	-0,383	-0,364	**64**	0,267	0,189	**89**	1,266	1,278
**15**	-1,039	-0,974	**40**	-0,368	-0,360	**65**	0,315	0,212	**90**	1,388	1,370
**16**	-0,985	-0,929	**41**	-0,365	-0,322	**66**	0,332	0,262	**91**	1,422	1,451
**17**	-0,960	-0,923	**42**	-0,316	-0,313	**67**	0,343	0,274	**92**	1,519	1,655
**18**	-0,904	-0,903	**43**	-0,314	-0,283	**68**	0,386	0,294	**93**	1,599	1,684
**19**	-0,850	-0,876	**44**	-0,298	-0,247	**69**	0,421	0,344	**94**	1,646	1,770
**20**	-0,835	-0,855	**45**	-0,269	-0,236	**70**	0,433	0,357	**95**	1,717	1,969
**21**	-0,805	-0,817	**46**	-0,253	-0,227	**71**	0,495	0,376	**96**	1,869	2,056
**22**	-0,787	-0,792	**47**	-0,199	-0,195	**72**	0,524	0,431	**97**	2,183	2,223
**23**	-0,763	-0,781	**48**	-0,172	-0,179	**73**	0,545	0,444	**98**	2,415	2,454
**24**	-0,738	-0,764	**49**	-0,128	-0,169	**74**	0,570	0,450	**99**	3,238	2,900

Normative data for ten neuropsychological tests for the Guatemalan pediatric population updated to account for vulnerability

Table S19. Transformation from values (z_i) to percentile scores in M-WCST task.

PC	M-WCST Cat.	Total error	Persev. err	PC	M-WCST Cat.	Total error	Persev. err	PC	M-WCST Cat.	Total error	Persev. err	PC	M-WCST Cat.	Total error	Persev. err
				**25**	-0,755	-0,851	-0,740	**50**	0,224	-0,151	-0,216	**75**	0,833	0,681	0,596
**1**	-3,004	-2,451	-1,508	**26**	-0,738	-0,831	-0,739	**51**	0,242	-0,112	-0,208	**76**	0,870	0,728	0,623
**2**	-2,273	-2,138	-1,424	**27**	-0,737	-0,797	-0,710	**52**	0,258	-0,070	-0,163	**77**	0,872	0,748	0,676
**3**	-2,112	-1,934	-1,361	**28**	-0,663	-0,788	-0,707	**53**	0,289	-0,025	-0,150	**78**	0,899	0,763	0,739
**4**	-1,875	-1,802	-1,260	**29**	-0,591	-0,749	-0,699	**54**	0,335	0,011	-0,142	**79**	0,907	0,814	0,771
**5**	-1,644	-1,685	-1,223	**30**	-0,509	-0,708	-0,670	**55**	0,349	0,032	-0,142	**80**	0,915	0,860	0,818
**6**	-1,581	-1,674	-1,145	**31**	-0,505	-0,695	-0,646	**56**	0,369	0,067	-0,124	**81**	0,941	0,904	0,833
**7**	-1,483	-1,578	-1,127	**32**	-0,399	-0,664	-0,635	**57**	0,378	0,092	-0,005	**82**	0,952	0,973	0,858
**8**	-1,483	-1,562	-1,107	**33**	-0,326	-0,650	-0,632	**58**	0,403	0,126	-0,004	**83**	1,032	1,046	0,955
**9**	-1,434	-1,508	-1,041	**34**	-0,312	-0,609	-0,621	**59**	0,403	0,170	0,032	**84**	1,032	1,077	0,967
**10**	-1,409	-1,411	-1,032	**35**	-0,226	-0,575	-0,596	**60**	0,417	0,180	0,095	**85**	1,032	1,146	1,016
**11**	-1,337	-1,395	-1,030	**36**	-0,226	-0,558	-0,572	**61**	0,487	0,208	0,112	**86**	1,032	1,301	1,066
**12**	-1,293	-1,320	-1,030	**37**	-0,184	-0,547	-0,509	**62**	0,494	0,255	0,149	**87**	1,062	1,357	1,111
**13**	-1,266	-1,283	-1,030	**38**	-0,184	-0,506	-0,490	**63**	0,494	0,293	0,201	**88**	1,142	1,464	1,195
**14**	-1,265	-1,263	-1,012	**39**	-0,170	-0,489	-0,463	**64**	0,540	0,321	0,209	**89**	1,142	1,590	1,269
**15**	-1,143	-1,235	-0,985	**40**	-0,127	-0,486	-0,463	**65**	0,540	0,367	0,246	**90**	1,158	1,651	1,385
**16**	-1,111	-1,189	-0,955	**41**	-0,061	-0,433	-0,415	**66**	0,573	0,444	0,276	**91**	1,209	1,809	1,551
**17**	-1,020	-1,156	-0,906	**42**	-0,053	-0,394	-0,369	**67**	0,641	0,481	0,288	**92**	1,297	1,841	1,608
**18**	-0,996	-1,107	-0,896	**43**	-0,022	-0,353	-0,365	**68**	0,685	0,520	0,383	**93**	1,297	1,870	1,674
**19**	-0,881	-1,040	-0,884	**44**	0,039	-0,341	-0,332	**69**	0,695	0,523	0,407	**94**	1,336	2,003	1,839
**20**	-0,881	-1,028	-0,857	**45**	0,086	-0,337	-0,290	**70**	0,701	0,545	0,407	**95**	1,336	2,061	1,947
**21**	-0,881	-1,014	-0,826	**46**	0,094	-0,313	-0,287	**71**	0,701	0,566	0,423	**96**	1,336	2,421	2,125
**22**	-0,879	-0,981	-0,821	**47**	0,102	-0,271	-0,264	**72**	0,701	0,596	0,505	**97**	1,345	2,500	2,321
**23**	-0,854	-0,944	-0,806	**48**	0,137	-0,164	-0,246	**73**	0,748	0,628	0,534	**98**	1,416	2,850	2,614
**24**	-0,853	-0,890	-0,765	49	0,182	-0,156	-0,236	**74**	0,783	0,637	0,579	**99**	1,479	3,558	3,252
